# Upcycling Waste Low‐Density Polyethylene into Highly Crystalline Graphite

**DOI:** 10.1002/advs.202416978

**Published:** 2025-04-11

**Authors:** Woojae Jeong, Haneul Nam, Hwansoo Shin, Sooin Hwang, Junho Lee, Dongho Lee, Hyeong Jun Kim, Jingi Ahn, Tae Hee Han

**Affiliations:** ^1^ Department of Organic and Nano Engineering Human‐Tech Convergence Program Research Institute of Industrial Science Hanyang University Seoul 04763 Republic of Korea; ^2^ Hyundai Motor Company Materials Research & Engineering Center Sustainable Materials Research Team Uiwang 16082 Republic of Korea

**Keywords:** graphene oxide, highly crystalline graphite, low‐density polyethylene (LDPE), stabilization process, upcycling, waste plastic

## Abstract

Only a small fraction of the global plastic waste is recycled, and the recycled materials are often of lower quality. For example, only 14% of the world's total plastic is recycled, with low‐density polyethylene (LDPE) accounting for only 4% of it. This study presents a novel approach to transforming waste LDPE into electrically conductive artificial graphite with high crystallinity using stabilization and graphitization processes. This method achieves a carbon yield of 89.4%, surpassing yields from conventional polymer precursors, such as polyacrylonitrile and polyamide. The resulting LDPE‐derived graphite (LGP) exhibits a high crystallinity and electrical conductivity, twice those of natural graphite and comparable to those of artificial graphite, which typically requires extensive heat treatment to prepare. LGP can be used in pastes and inks for advanced applications, such as 3D printing, flexible electrodes, heaters, and photothermal devices. Additionally, the high crystallinity of LGP enables the formation of larger graphene oxide (GO) with an average size of 4.1 ± 2.4 µm, because the larger crystalline domains in LGP facilitate the exfoliation process. The approach developed in this study contributes significantly to LDPE waste management and provides a pathway for the fabrication of valuable carbon materials for broad industrial applications.

## Graphical abstract

This study achieved high‐crystallinity graphite from low‐density polyethylene (LDPE) and LDPE products, offering 89.4% carbon yield. Enhanced conductivity and crystallinity support applications in electronics, 3D printing, and photothermal devices. Efficient graphene oxide production demonstrates LDPE‐derived graphite (LGP)'s value in various applications addressing environmental applications, addressing plastic waste challenges by transforming LDPE into valuable carbon materials.

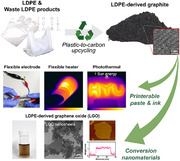

## 1 Introduction

**Figure 1:**
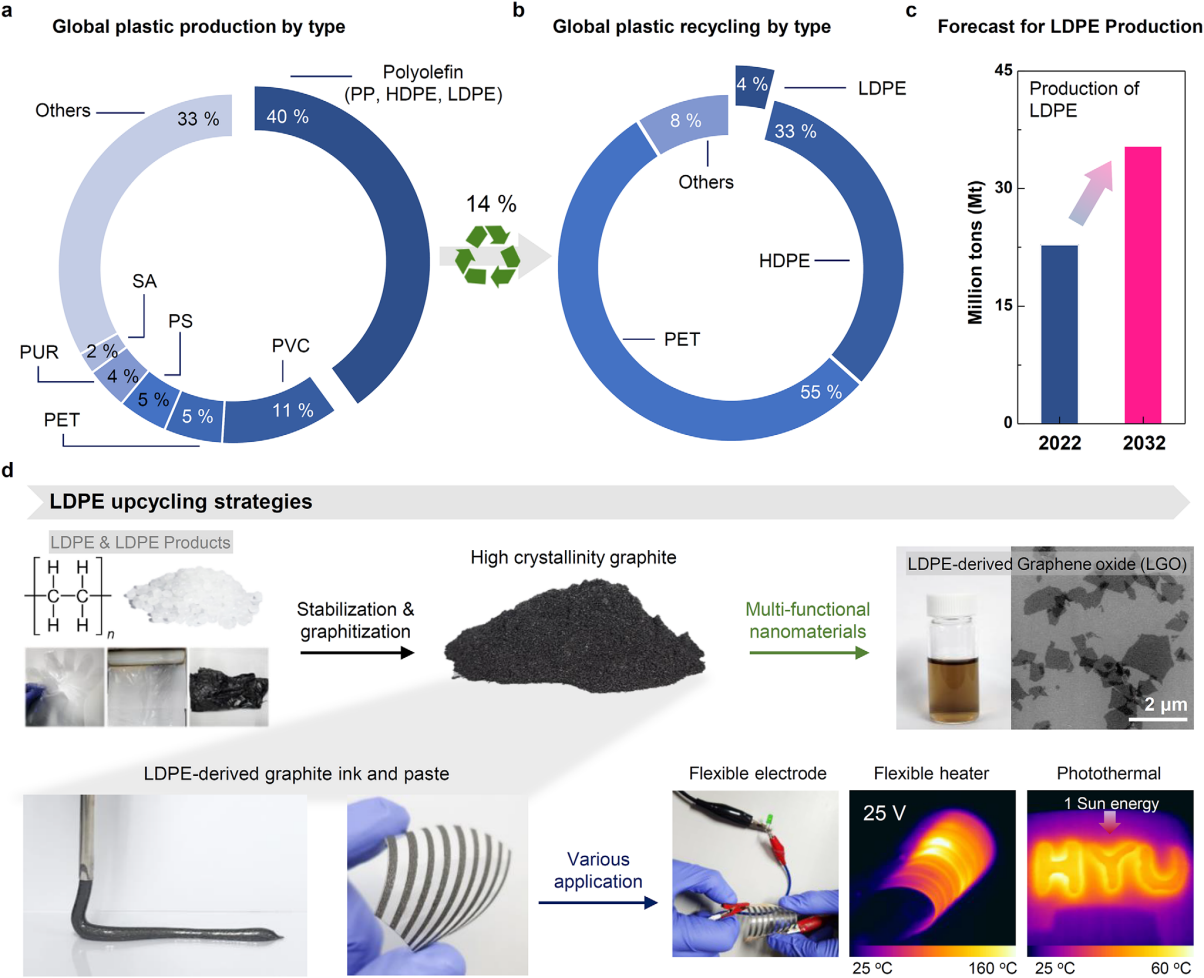
a) Global distribution of plastic types and the percentage of total recycled products; only ≈14% of the total plastic materials are recycled. b) Recycling rates of different plastic types worldwide in 2017 (PP: polypropylene, HDPE: high‐density polyethylene, LDPE: low‐density polyethylene, PVC: polyvinyl chloride, PET: polyethylene terephthalate, PS: polystyrene, PUR: polyurethane, SA: styrene acrylonitrile). c) Production volume of LDPE in 2022 and projected production volume in 2032. d) Applications of highly crystalline graphite prepared from LDPE in 3D printer pastes and inks, flexible electrodes, heaters, and photothermal films, and its use as graphene oxide.

Plastic waste accumulation, driven by increasing production and consumption in recent decades, has become a growing global challenge. This issue has severe environmental consequences, because marine pollution and conventional waste management methods, such as landfilling and incineration, significantly contribute to CO_2_ emissions and other greenhouse gases.^[^ ^1^, ^2^, ^3^, ^4^ ^]^ These practices accelerate climate change, contaminate soil and water, and disrupt ecosystems. Among the different types of plastic waste, polyolefins—mainly polyethylene (PE) and polypropylene (PP)—constitute the largest proportion (**Figure** **1**a).^[^ ^5^, ^6^ ^]^ Despite the widespread use of polyolefins, their overall recycling rate remains low at ≈14%. In particular, low‐density polyethylene (LDPE) is challenging to recycle, because it is primarily used in film‐type products, such as thin films, wire coatings, and disposable items, which degrade easily upon surface exposure, making them more susceptible to environmental factors. Recycled LDPE tends to have poor mechanical properties, which limit its reuse in applications requiring strength and durability, such as films and disposable items. Consequently, LDPE accounts for only 4% of all recycled plastics, which is substantially lower than those of other polyolefins, such as high‐density polyethylene (HDPE) and PP (Figure 1b).^[^ ^7^, ^8^, ^9^ ^]^ Despite these barriers, global LDPE production continues to increase, and its production reached 22.8 million tons in 2022 and is projected to increase to 35.4 million tons by 2030 (Figure 1c).^[^ ^10^ ^]^ These trends highlight the urgent need for innovative upcycling solutions to manage LDPE waste and unlock its potential for high‐value applications.

The effective management of polymer waste relies on thermochemical processes that are commonly used for plastic waste treatment, such as pyrolysis^[^ ^11^ ^]^ and gasification.^[^ ^12^ ^]^ Anaerobic carbonization, a cost‐effective method, involves the high‐temperature treatment (>800 °C) of polymers in an inert environment to convert them into carbon materials. However, polyolefins start to decompose at relatively low temperatures (400–500 °C),^[^ ^13^ ^]^ producing volatile by‐products, such as hydrocarbon gases, instead of forming solid carbon structures. This premature decomposition limits the extent of aromatization, which is a critical step in the formation of solid carbon, and ultimately reduces the carbon yield.^[^ ^13^ ^]^ To address this limitation, stabilization is essential to prevent the premature decomposition of polyolefins, thereby allowing them to undergo structural transformations that facilitate solid carbon formation during the carbonization process. Processes such as thermal oxidation introduce oxygen functionalities, which induce the formation of cyclic ladder‐like structures that link the polymer backbone, thereby enhancing the stability of the intermediates and facilitating aromatization. This approach can boost the carbon yield to ≈50% for PE, which otherwise does not produce solid carbon in inert atmospheres (e.g., argon or nitrogen).^[^ ^14^ ^]^ However, these methods primarily stabilize only the polymer surface, and the interior of the material is barely stabilized, which remains a significant obstacle to further increasing the overall carbon yield.^[^ ^15^ ^]^

Herein, we present an effective method for converting LDPE into highly crystalline graphite, achieving high carbon yields and well‐ordered carbon structures. The process involves the hydrothermal treatment of LDPE in a mixture of HCl and HNO_3_ at temperatures up to 300 °C for stabilization (denoted as HN stabilization), followed by high‐temperature graphitization at 2700 °C. This method enhances the formation of stable carbon backbones, resulting in a graphite material with superior electrical conductivity. The optimized HN stabilization provides 89.4% carbon yield, which is higher than that obtained from traditional polymer precursors such as polyacrylonitrile (PAN) and polyimide (PI), where carbon yield refers to the yield of carbon char relative to the fixed carbon content in LDPE. The LDPE‐derived graphite (LGP) exhibits twice the crystallinity and electrical conductivity of natural graphite while offering a performance comparable to that of artificial graphite, which typically requires extensive heat treatment. Furthermore, LGP can be converted into GO using a modified Hummers’ method, which enables the production of conductive pastes and inks for advanced applications, including 3D printing, flexible electronics, and photothermal devices (Figure 1d). Importantly, our process is effective for both pure and waste LDPE samples and delivers a consistently high performance. This approach provides a sustainable method for upcycling plastic waste. In addition, it contributes to the production of high‐value carbon materials for broad industrial applications, particularly in energy storage and electronic devices.

## 2 Results and Discussion

### 2.1 Stabilization of LDPE to Obtain Highly Crystalline Graphite

The chemical and environmental instabilities of LDPE often result in the formation of free radicals through chain scission; the free radicals readily react with oxygen, resulting in the degradation of its mechanical and optical properties. However, under controlled oxidation conditions, linear PE chains can be crosslinked with oxygen atoms to form stable cyclic ladder structures at elevated temperatures. This transformation, termed stabilization, enables the successful carbonization of LDPE into a solid carbonaceous material. In particular, pure LDPE rapidly decomposes ≈400 °C, resulting in complete weight loss, indicating its inability to independently transform into a carbon structure. Differential scanning calorimetry (DSC) measurements revealed that the glass transition (*T*_g_) and melting temperatures (*T*_m_) of LDPE are ≈100 and 120 °C, respectively (Figure S1, Supporting Information). Notably, LDPE transitions to a liquid state before carbonization, unlike traditional carbon precursors such as PAN^[^ ^16^, ^17^, ^18^ ^]^ and PI,^[^ ^19^ ^]^ which can be stabilized at high temperatures without their melting because of their strong intermolecular interactions and rigid structures. The liquid‐phase formation of LDPE, observed at ≈120 °C, allows it to undergo more uniform reactions than solid‐state materials, thereby enhancing the efficiency of the stabilization process.^[^ ^20^ ^]^ In the case of PAN‐based carbon fibers, oxygen functionalities are introduced from atmospheric oxygen during stabilization; however, nonuniform oxygen diffusion often induces phase differences between the shell and core regions. These diffusion‐related instabilities create inconsistencies in the cyclic ladder structures, which can disrupt the uniform stacking and alignment of carbon layers during the subsequent graphitization process, ultimately resulting in a less crystalline structure. However, the melting behavior of LDPE enables more uniform oxygen diffusion, thereby minimizing these inconsistencies and facilitating a more homogeneous carbon structure after graphitization.

To assess the stabilization efficiency of LDPE in comparison with those of other polyolefins, we applied three distinct stabilization treatments on LDPE, HDPE, and PP: the HN stabilization method, a hydrothermal reaction with sulfuric acid, and oxygen atomistic oxidation. To maintain an objective comparison, all stabilization processes were carried out at a fixed temperature of 250  °C for a consistent duration of 12 h. Following stabilization, the product was carbonized at 800 °C in an Ar atmosphere. This allowed us to measure the lateral crystallite size (*L*_a_) and stacking height (*L*_c_) of the resultant carbon crystals. *L*_a_, which represents the crystallite size within the disordered and amorphous carbon regions, was determined via Raman spectroscopy using the Ferrari–Robertson relationship.^[^ ^21^ ^]^ *L*_c_, which indicates the vertical alignment of the graphene layer, was determined by the Scherrer analysis of the (002) diffraction peak width. As shown in Table S1 (Supporting Information), HN‐stabilized LDPE exhibited the highest crystallinity among the samples. Its high crystallinity can be attributed to the distinct interaction between the branched molecular structure of LDPE and the HN stabilization process, which facilitates consistent oxygen diffusion throughout the polymer matrix, facilitating a more controlled reaction. This consistency reduces the structural variability observed in HDPE and PP, for which the disordered regions often interrupt the formation of well‐aligned cyclic ladder structures. Realizing the homogeneous stabilization of the polymer before its graphitization is essential because it reduces irregularities in the carbon layer stacking and promotes a highly ordered carbon framework. The results in Table S1 (Supporting Information) highlight the effectiveness of HN stabilization in converting LDPE into a highly crystalline carbon material.

**Figure 2:**
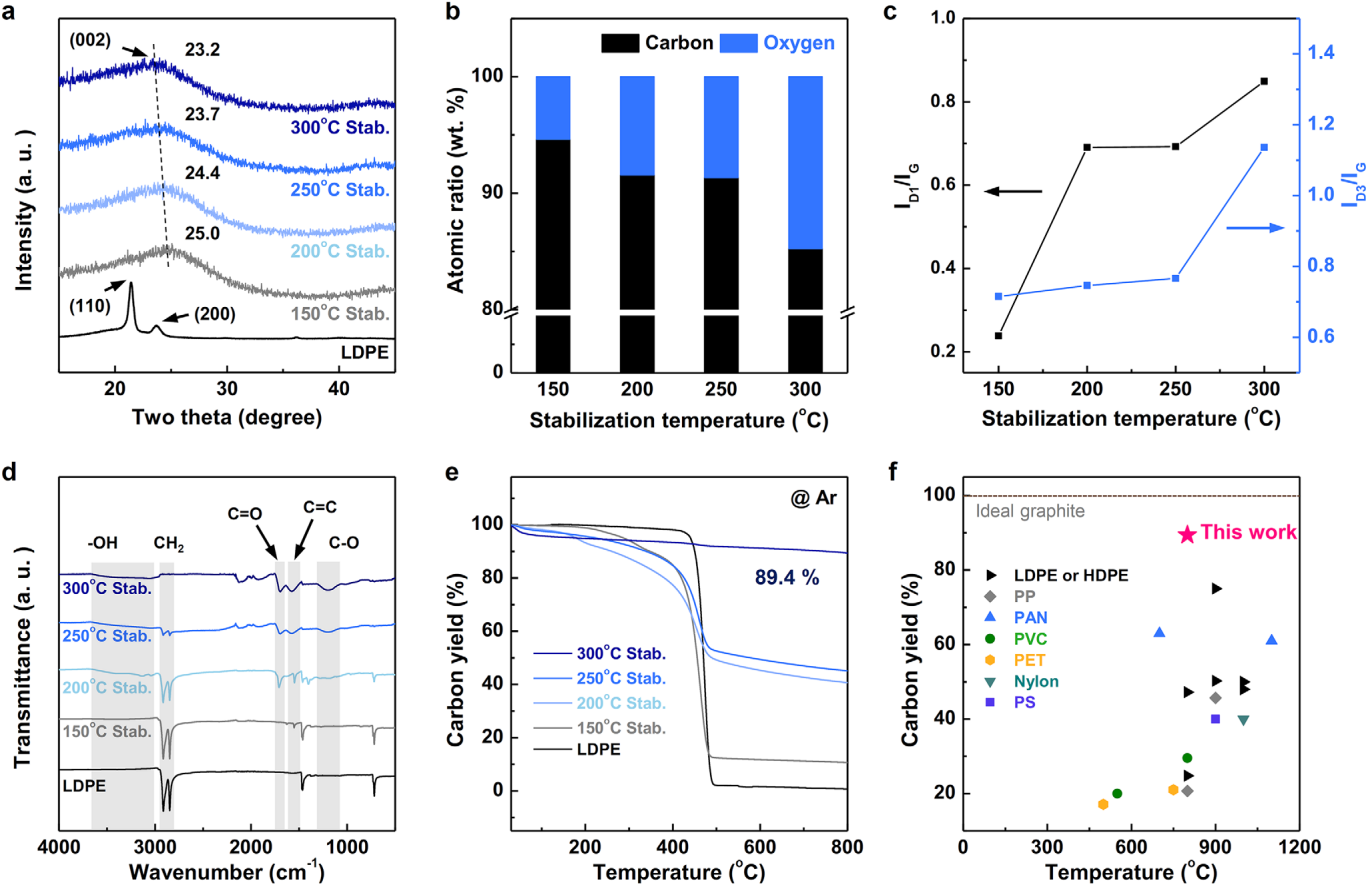
a) XRD patterns of LDPE subjected to HN stabilization at different temperatures, b) atomic ratio determined by XPS analysis, c) *I*_D1_/*I*_G_ and *I*_D3_/*I*_G_ ratios obtained by fitting the Raman spectra, d) FT‐IR spectra, e) TGA curves, and f) Ashby plot of the carbon yield of various polymers pretreated using different methods for carbon production.

Various temperature conditions were explored to further optimize the HN stabilization process. **Figure** **2**a presents the X‐ray diffraction (XRD) patterns of pristine LDPE and the LDPE samples stabilized using the HN process at 150, 200, 250, and 300 °C. Pure LDPE initially exhibited distinct peaks corresponding to its (110) and (200) planes, indicative of orthorhombic LDPE crystals.^[^ ^22^, ^23^ ^]^ Upon HN stabilization at 150 °C, these peaks disappeared, and a (002) peak began to appear. This (002) peak is characteristic of carbonized structures found in materials such as lignin, reduced graphene oxide, and stabilized PAN fibers, suggesting the onset of carbonaceous ordering within the LDPE matrix.^[^ ^24^, ^25^ ^]^ As the stabilization temperature was increased from 150 to 300 °C, the (002) peak shifted from 2*θ* = 25.0° to 23.2°, indicating an increase in *d*‐spacing. X‐ray photoelectron spectroscopy (XPS) confirmed that the increased *d*‐spacing was due to the incorporation of oxygen functional groups into LDPE during HN stabilization (Figure 2b). These oxygen atoms disrupt the regular arrangement of the LDPE chains by creating crosslinks, thereby increasing the spacing between the carbon planes. To further investigate the chemical transformations during stabilization, we conducted a detailed XPS C 1s spectral analysis on LDPE samples treated at different temperatures. The results showed a gradual decrease in the C─C peak (284.5 eV) and a corresponding increase in oxygen‐containing functional groups, such as C─OH, C─O (286.3 eV), and O═C─O (289.7 eV), with the O═C─O peak appearing at 250 °C (Figure S2, Supporting Information). Elemental analysis further confirmed these trends, showing an increase in oxygen content alongside a rise in carbon content as the stabilization temperature increased, which was consistent with the XPS findings (Table S2, Supporting Information). Raman analyses revealed the presence of characteristic D and G bands in the carbon structure of LDPE, confirming the structural reorganization induced by the oxygen functional groups.^[^ ^14^, ^26^, ^27^ ^]^ This result suggests that HN stabilization facilitates uniform reactions that generate aromatic hydrocarbon structures in LDPE. As the HN stabilization temperature increased, both the ratio of the D1 band (disordered graphite lattices) to G band (ideal graphitic lattice) and that of the D3 band (amorphous carbon) to G band increased, consistent with the trends observed in the XRD analyses (Figure 2c).^[^ ^28^ ^]^ These findings demonstrate the importance of optimizing the stabilization conditions to enhance the crystallinity and uniformity of carbon structures derived from LDPE and provide valuable insights for developing high‐performance carbon materials.

Additionally, Fourier transform infrared (FT‐IR) spectroscopy revealed the transformation of the LDPE chains and the formation of oxygen functional groups at different HN stabilization temperatures (Figure 2d). Pure LDPE showed typical peaks at 717, 1470, 2846, and 2912 cm^−1^_,_ corresponding to the rocking vibration, bending deformation, and symmetric and asymmetric stretching of the ─CH_2_ groups, respectively.^[^ ^29^ ^]^ When LDPE was stabilized at 150 °C, weak peaks of certain functional groups such as C═C (1600 cm^−1^) and C═O (1700 cm^−1^) appeared, indicating the local oxidation of the polymer chains. The transformation progressed further as the stabilization process was continued at higher temperatures. At 250 °C, significant changes in the functional groups were observed, and at 300 °C, the characteristic peaks of LDPE were no longer visible, suggesting extensive structural reorganization in the material. However, the simultaneous increase in the intensities of the C─O (1250 cm^−1^), C═C (1600 cm^−1^), C═O (1700 cm^−1^), and ─OH (3200 cm^−1^) functional groups clearly indicated that the polymer chains were reorganized into a stable carbon network without undergoing decomposition. Increasing the stabilization temperature further to 320 °C did not produce noticeable spectral changes, suggesting that the transformation was complete at 300 °C (Figure S3, Supporting Information). This structural evolution is critical, because it stabilizes LDPE at high temperatures, preparing it for subsequent graphitization.

The structural transformation of LDPE was further confirmed by TGA, which indicated a substantial increase in the carbon yield from 45.2% at 250 °C to 89.4% at 300 °C, suggesting that the stabilization process induced significant molecular reorganization (Figure 2e). This high carbon yield reflects the effectiveness of the stabilization process, which facilitates increased crosslinking and the incorporation of oxygen functionalities, owing to which the carbon content is retained within the polymer matrix and the release of volatile byproducts is minimized. The retention of carbon at elevated temperatures facilitates efficient graphitization, enabling the production of a highly crystalline carbon structure for high‐performance applications.

FT‐IR spectroscopic and TGA analyses suggested that C─O bonds play a key role in transforming the LDPE chains into cyclic ladder structures. The introduction of oxygen functional groups drives the crosslinking of the polymer chains, which is essential for the formation of cyclic ladder structures. The crosslinking of polymer chains restricts chain mobility and promotes the formation of condensed aromatic structures during carbonization. The ordered arrangement resulting from the oxygen linkages enhances the graphitization process, promoting the formation of highly crystalline carbon frameworks. Cross‐linked networks are important for converting carbon structures, preventing excessive structural degradation, and enabling higher yields of graphitic carbon.^[^ ^30^ ^]^ To further illustrate the temperature‐dependent incorporation of oxygen functional groups, Figure S4 (Supporting Information) presents a schematic representation of the progressive transformation of LDPE during HN stabilization. Figure 2f compares the carbon yields of various polymers stabilized using different methods in earlier studies.^[^ ^14^, ^31^, ^32^, ^33^, ^34^, ^35^, ^36^, ^37^, ^38^, ^39^, ^40^, ^41^, ^42^, ^43^, ^44^, ^45^ ^]^ This study demonstrates that the HN stabilization of LDPE can serve as an efficient pretreatment process for producing artificial graphite by forming C─O bonds during stabilization, thereby demonstrating the potential of LDPE as a precursor for carbon materials.

### 2.2 Thermal Annealing‐Induced Structural Transformation and Crystallinity Enhancement in HN‐Stabilized LDPE

**Figure 3:**
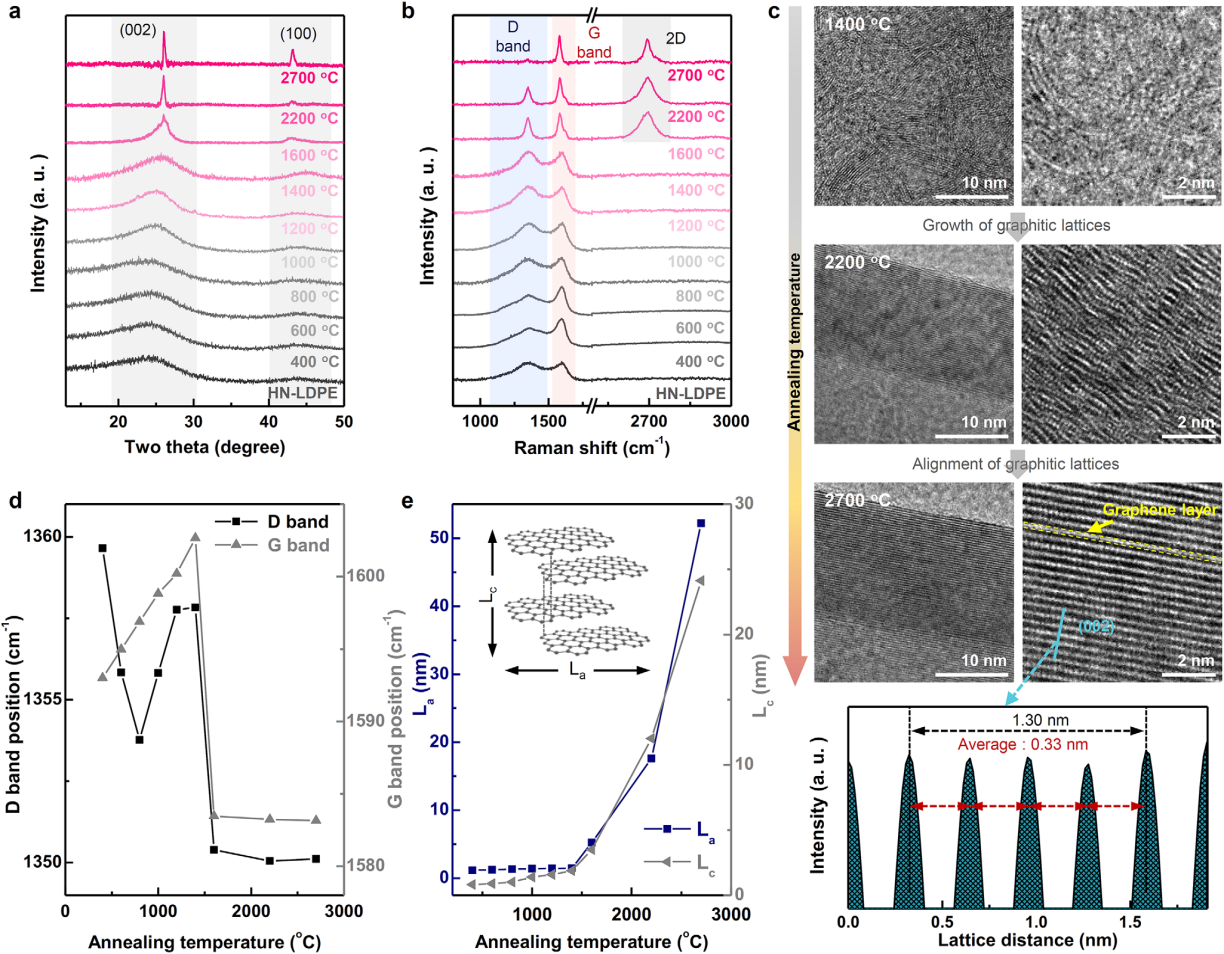
a) XRD patterns and b) Raman spectra of HN‐LDPE obtained after thermal annealing at different temperatures in the 400–2700 °C range. c) TEM images of the samples annealed at 1400, 2200, and 2700 °C, and the FFT data of the sample annealed at 2700 °C. d) D and G band positions, and e) basic structural unit (BSU) growth data of HN‐LDPE samples after carbonization and graphitization under Ar at different temperatures.

After the stabilization process, the HN‐stabilized LDPE was thermally annealed at 300 °C (HN‐LDPE) in an inert (Ar) atmosphere. XRD analyses revealed significant changes in the (002) peak of HN‐LDPE over the temperature range of 400–2700 °C (**Figure** **3**a; Figure S5, Supporting Information). As the annealing temperature increased, the (002) peak became progressively sharper and more symmetrical, resulting in a significant reduction in its full width at half‐maximum (FWHM). These observations suggest a transition from a disordered to an ordered carbon structure; they with continuous crystal growth as the temperature increased. As the annealing temperature was increased to ≈1400 °C, the (002) peak exhibited a distinct asymmetry, which is characteristic of the turbostratic structure observed in anthracite.^[^ ^45^ ^]^ The broad and unsplit nature of the (100) band suggests that the material retained a polycyclic aromatic hydrocarbon structure that is typical of anthracite. This result indicates that carbonization at these temperatures produces a layered carbon structure with significant disorder, suggesting that the graphene layers remained randomly stacked, lacking long‐range order. As the annealing temperature exceeded 1600 °C, denoting the beginning of graphitization, the (002) peak became notably stronger and narrower, demonstrating enhanced structural ordering and increased graphite crystallinity. The peak sharpening and FWHM reduction reflect the growth of larger graphite crystallites and a decrease in structural defects. This transformation suggests that graphitization at higher temperatures facilitates the alignment and stacking of the graphene layers, resulting in a more crystalline graphitic structure. These structural changes provide essential insights into the carbonization and graphitization processes, as observed in the evolution of the (002) peak. The (002) peak evolution in the XRD patterns indicates significant atomic‐level reorganization within the material. Initially, a turbostratic structure was formed owing to the random orientation of the polycyclic aromatic hydrocarbons. However, as the temperature increased, sufficient energy was supplied for these structures to rearrange into a more ordered form, leading to the formation of crystalline graphite at 2700 °C.

Not all carbon precursors can produce highly ordered graphitic carbon during high‐temperature graphitization. Carbon materials can be classified as graphitizable or non‐graphitizable carbon, referred to as soft or hard carbon, respectively. Only soft carbon can undergo the necessary long‐range order and graphite layer stacking. The turbostratic structure of the hard carbon remains disordered; it does not evolve into a highly ordered graphite structure, even after high‐temperature treatment.

The degree of alignment is closely related to the mechanical strength and electrical conductivity of the material, which are critical for specific applications. To gain deeper insights into the structural changes in HN‐stabilized LDPE, XRD analysis was complemented by Raman and TEM analyses (Figure 3b–d). The characteristic Raman peaks of carbon materials in the 1050–1730 cm^−1^ range are indicative of distinct structural features and defect types in carbon, and are classified as D1 (≈1350 cm^−1^), D2 (≈1620 cm^−1^), D3 (≈1500 cm^−1^), D4 (≈1200 cm^−1^), and G (≈1580 cm^−1^) bands.^[^ ^28^ ^]^ To simplify the analysis, we grouped the peaks in the 1050–1450 cm^−1^ region of the Raman spectrum as the D band associated with disorder and those in the 1500–1730 cm^−1^ region as the G band related to graphitization. Variations in the D and G band positions and their relative intensities reflect shifts in the defect density and crystallinity levels during thermal annealing. Specifically, a shift in the D band represents a change in disorder, whereas a shift in the G band indicates the degree of graphitic ordering achieved by the thermal treatment of the carbon precursor. This analysis provides insights into the structural evolution of the material during thermal annealing.

The structural transformation of HN‐LDPE was assessed by analyzing the shifts in the D and G bands. The annealing temperatures were categorized into four distinct regions: 400–800 °C (AT1), 800–1400 °C (AT2), 1400–2200 °C (AT3), and 2700 °C (AT4). In the AT1 region, disordered carbon structures were formed owing to the thermal transformation of HN‐LDPE, accompanied by an increase in the amorphous carbon content. The shift in the D band to a lower frequency indicates increased disorder caused by accumulated defects. In contrast, the shift in the G band to a higher frequency suggests stress from the newly formed amorphous carbon. In the AT2 region, the D band shifted to a higher frequency, indicating reduced disorder; this is because the defects were rearranged and partially eliminated. Moreover, the carbon structure began to realign into a sp^2^‐carbon network, as evidenced by the shift in the D‐band position.^[^ ^45^, ^46^ ^]^ Cross‐sectional TEM images of the carbon structures obtained by annealing HN‐LDPE at 1400 °C showed a random orientation of crystalline carbon and the coexistence of amorphous carbon. In the AT3 region, the D and G bands were stabilized at ≈1350 and 1583 cm^−1^, respectively, and the 2D peak became more pronounced, reflecting the formation of ordered graphitic structures. Although the defects in the graphene layers and misalignments in the graphitic lattices were retained, the reduction in the defect density suggests a transition toward greater crystallinity. Nearly all defects were eliminated at 2700 °C (AT4), and the carbon structure was fully aligned, resulting in highly crystalline graphite. The appearance of the prominent 2D band at this stage confirms the formation of well‐ordered graphene layers. Further, the fast‐Fourier transform (FFT) analysis of the TEM image revealed a layer‐to‐layer spacing of 0.33 nm, which is consistent with the formation of high‐quality graphite. This material (annealing temperature: 2700 °C) is hereafter referred to as LGP. To analyze the structural evolution of the carbon precursor during annealing, the dimensions of the structural units, specifically *L*_a_ and *L*_c_, were calculated after annealing treatment at various temperatures (Figure 3e). For samples annealed at temperatures ranging up to 1400 °C, owing to the presence of disordered and amorphous carbon, the Ferrari–Robertson relation was applied to estimate these dimensions. For samples annealed at temperatures exceeding 1600 °C, for which the 2D band appeared, the Tuinstra–Koenig relation was applied. In the HN‐LDPE samples, the *L*_a_ and *L*_c_ values were initially 1.2 and 0.8 nm, respectively, and they gradually increased to 1.5 and 1.4 nm after being annealed at 1400 °C, indicating a slow crystallization process. When annealed at ≥1600 °C, *L*_a_, and *L*_c_ increased rapidly and reached 5.2 and 3.5 nm, respectively, and ultimately 52.2 and 24.1 nm at 2700 °C. This rapid growth can be attributed to enhanced atomic mobility, defect elimination, and improved sp^2^ bonding at higher temperatures. At these elevated temperatures, the oxygen functional groups react with carbon to form CO or CO_2_, which escapes as a gas, leaving behind a pure carbon structure.

### 2.3 Conversion of LGP into Nanomaterials and its Applications in Paste and Ink

**Figure 4:**
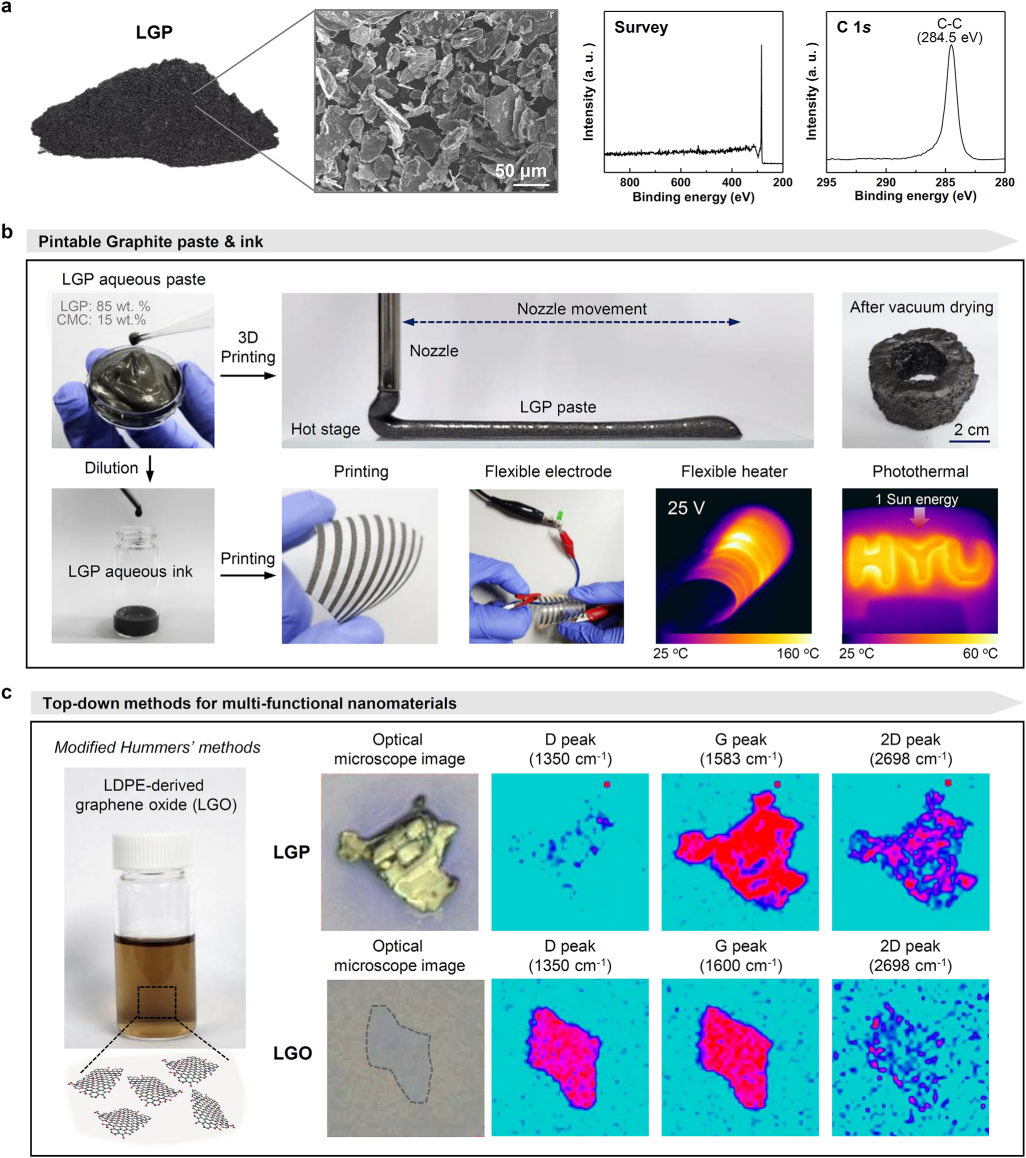
a) Photograph, SEM image, XPS survey, and C 1s profiles of the produced LGP. b) Photograph of the LGP aqueous paste being used in 3D printing, and photographs illustrating various applications of the LGP aqueous ink. c) Photograph of the aqueous suspension of LGO derived from LGP, and 2D Raman maps of LGP and LGO.

The potential of LGP in various applications was explored. **Figure** **4**a shows a photograph and scanning electron microscopy (SEM) image of LGP, and the XPS analysis reveals that it consists purely of carbon, without other elemental impurities. Figure 4b illustrates the use of LGP in aqueous pastes and inks. To address the irregular surfaces and surface‐charged groups of the LGP powder, 15 wt.% carboxymethyl cellulose (CMC) was added as a dispersant, which resulted in an LGP paste. The application of this improved paste in 3D printing was successfully verified. The paste retained its shape when extruded through a nozzle, enabling the high‐precision and detailed expression of the 3D‐printed structures. In addition to its applicability in 3D printing, LGP ink has significant potential for fabricating various flexible electronic components. The flexible electrodes and heaters printed with the LGP ink performed consistently during testing, even under bending and deformational stresses. This result indicates that the LGP ink maintains its electrical conductivity and thermal functionality, which makes it highly suitable for flexible electronic devices that require mechanical flexibility.

Furthermore, LGP serves as a precursor for GO, a multifunctional nanomaterial known for its versatility in various applications. A single‐layered GO was successfully exfoliated from LGP using a modified Hummers method that has been widely adopted in research laboratories.^[^ ^47^, ^48^ ^]^ Hereafter, the GO derived from LGP is referred to as LGO. The LGO synthesized in this study dispersed well in deionized water (DIW), and the topography observed in atomic force microscopy (AFM) revealed nanosheets with heights of ≈1.2 nm (Figure S6a, Supporting Information). SEM analyses revealed an average diameter of 4.1 ± 2.4 µm, consistent with that of a typical GO nanosheet (Figure S6b, Supporting Information). Comparative Raman mapping of LGP and LGO was performed to evaluate the synthetic quality by analyzing the characteristic peaks, defects, impurities, uniformity, and degree of oxidation of the carbon structures. The D, G, and 2D bands of carbon were investigated. As shown in Figure 4c, whereas strong G and 2D bands were observed for most areas, the D band of LGP was detected for the surface edges. In contrast, LGO exhibited strong intensities of both the D and G bands, indicating a uniform oxidation reaction throughout the nanosheet, thereby confirming the synthesis of high‐quality GO. XPS, FT‐IR spectroscopy, and Raman spectroscopy were used to compare the surface chemical and structural differences between LGO obtained from LGP and GO obtained from artificial graphite (Figure S7a–d, Supporting Information). The LGO produced in this study exhibited chemical and structural similarities to GO derived from artificial graphite, indicating its potential as a substitute for such materials.

### 2.4 Evaluating the Potential of Waste LDPE for High‐Quality Graphite Production

**Figure 5:**
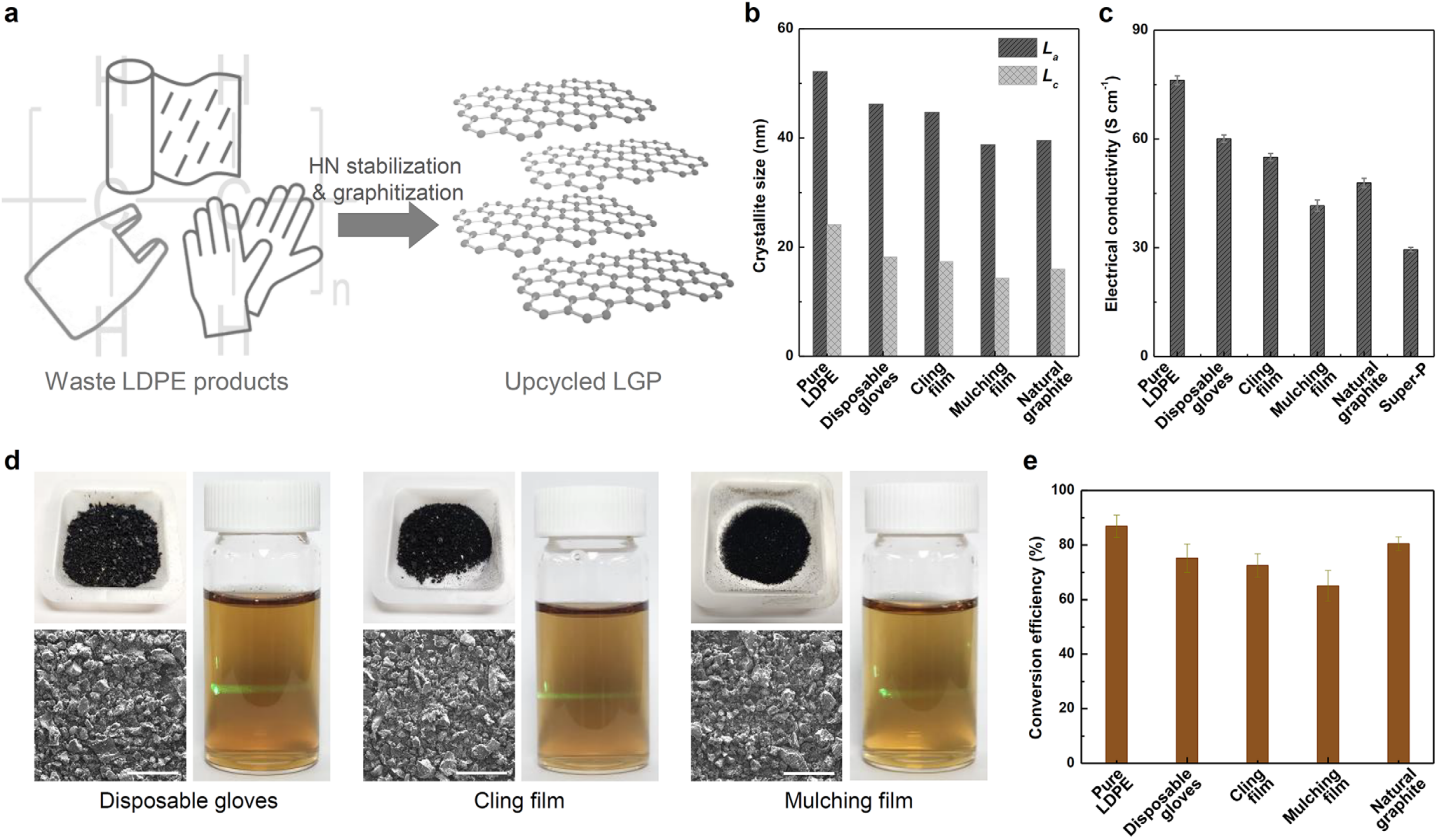
a) Potential for high‐crystallinity graphitization of various waste LDPE products, b) basic structural unit and c) electrical conductivity after HN stabilization and graphitization, d) photographs and SEM images of the LGP samples obtained from waste LDPE products, and images of LGO dispersions in water, e) conversion efficiency to graphene oxide for LGP derived from pure LDPE, LGP derived from waste LDPE products, and natural graphite.

The transformation of waste LDPE into graphite represents a promising strategy for addressing both plastic waste recycling and the environmental concerns associated with landfilling and incineration. To evaluate the efficacy of the thermal oxidative stabilization of waste plastics as an upcycling method, various waste LDPE products (disposable gloves, cling films, and agricultural mulch films) were subjected to HN stabilization, followed by thermal annealing (**Figure** **5**a; Figure S8, Supporting Information). Raman spectroscopy and XRD analyses provided detailed insights into the structural changes, revealing that the *L*_a_ values for pure LDPE, disposable gloves, cling films, mulch films, and natural graphite were 52.2, 46.2, 44.7, 38.8, and 39.6 nm, respectively. The corresponding *L*_c_ values were 24.1, 18.2, 17.3, 14.3, and 16.0 nm, respectively (Figure 5b). Although the crystallite sizes of the recycled‐LDPE‐derived graphite were smaller than those of the pure LDPE‐derived graphite, they remained comparable to those of natural graphite. The lower crystallinity of the mulch film‐derived graphite can be attributed to the interference of the carbon black additive, which disrupts carbon growth and alignment during the graphitization process. Electrical conductivity measurements further confirmed the differences in the crystallite sizes. The conductivities of the graphite samples obtained from pure LDPE, disposable gloves, cling films, and mulch films were 76.2, 60.1, 55.0, and 41.6 S cm^−1^, respectively. The conductivity of natural graphite was 47.9 S cm^−1^. Notably, all these values exceed the conductivity of Super P (29.5 S cm^−1^), a material widely applied in batteries (Figure 5c). These findings indicate that despite the slightly lower crystallinity of the waste‐plastic‐derived graphite samples, they can be effectively upcycled into high‐crystallinity graphite through carbonization. The upcycled graphite has comparable electrical conductivity to that of natural graphite, substantiating the idea that waste LDPE can be repurposed into high‐value graphite materials. This study demonstrates that waste LDPE can serve as a precursor for carbon materials suitable for advanced applications, including flexible electronics, heaters, photothermal devices, and graphene oxide production.

SEM studies revealed that graphite derived from the three recycled LDPE products exhibited morphological similarities but had more irregular shapes than pure LDPE‐derived graphite (Figure 5d). This irregularity is likely due to chemical additives, such as plasticizers and adhesion promoters, present in LDPE, which may disrupt the structural rearrangement of the carbon chains during stabilization and graphitization. To fully understand these effects, further studies focusing on the isolation or removal of these additives are required. After synthesizing GO, we recovered the unreacted materials through centrifugation. These unreacted materials were then heat‐treated at 800 °C in an Ar atmosphere to remove any residual oxygen functional groups present in them (Figure S9, Supporting Information). The conversion efficiency of pure LDPE and recycled LDPE‐derived graphite to GO was determined by comparing the weight of the unreacted material before and after synthesis (Figure 5e). Pure LDPE showed a conversion efficiency of 86.9%, which is 6.4% higher than that of natural graphite, whereas disposable gloves, cling films, and mulch films exhibited lower efficiencies of 75.2, 72.5, and 65.1%, respectively. The LGO synthesized from these LGP products demonstrated good water dispersion and the Tyndall effect, indicating stable colloidal properties suitable for advanced applications. With further optimization of the synthesis, recycled LDPE products can be used as valuable sources for producing high‐quality graphene oxide, despite the presence of additives and impurities.

## 3 Conclusion

We developed an effective method of HN stabilization followed by graphitization for converting LDPE into highly crystalline and electrically conductive graphite. The HN stabilization process increased the carbon yield to 89.4%, which surpasses the carbon yields of the conventional polymer precursors. XRD, Raman spectroscopy, and FT‐IR spectroscopy analyses confirmed the formation of cyclic ladder structures during stabilization, which contributed to a high carbon yield and structural quality after thermal annealing. The resulting LGP exhibited ≈1.3 times higher crystallinity and 1.6 times higher electrical conductivity than those of natural graphite; moreover, its crystallinity and conductivity were comparable to those of synthetic graphite, which typically requires extensive heat treatment to prepare. In addition, LGP was formulated into a paste and ink, and its potential for 3D printing and fabricating flexible electrodes, heaters, and photothermal devices was demonstrated. Its high crystallinity also facilitates its conversion to GO using the Hummers method. Ultimately, this approach enhances the recycling potential of LDPE, while offering a sustainable route for producing high‐value carbon materials with the added benefits of reducing plastic waste and supporting environmental sustainability. This method holds significant promise for delivering economically viable materials for advanced technological applications.

## 4 Experimental Section

### Materials

LDPE (melt index: 25 g 10 min^−1^), sodium CMC (molecular weight: ≈250 000), and 99% potassium permanganate (KMnO_4_) were purchased from Sigma–Aldrich (St. Louis, MO, USA). Hydrochloric acid (HCl, 35%), sulfuric acid (H_2_SO_4_, 95%), and hydrogen peroxide (H_2_O_2_, 35%) were purchased from Junsei Chemicals (Japan). Acetone (99.5%) and nitric acid (68–70%) were obtained from Daejung Chemical (Republic of Korea). DI water was obtained using a water purification system (Direct Q3) from Millipore (Billerica, MA, USA).

### Stabilization of LDPE and LDPE Products

LDPE and LDPE products were stabilized under identical conditions. To oxidize LDPE and its products, 10 g of each product was mixed with a mixed solution of hydrochloric acid and nitric acid (1:2 molar ratio; 50 mL) and loaded into a Teflon‐lined autoclave. Hydrothermal synthesis was performed for 12 h at specified temperatures. The samples were then washed several times with excess DI water via vacuum filtration to remove residual acids and dried under vacuum at 80 °C to obtain a powder.

### Carbonization and Graphitization of LDPE and LDPE Products

The stabilized LDPE and the LDPE products were thermally annealed in Ar. The powder samples were placed in a graphite crucible and heated to 2000 °C at the rate of 5 °C min^−1^, then to 2500 °C at 3 °C min^−1^, and finally to 2700 °C at 2 °C min^−1^. The samples were held at the final temperature of 2700 °C for 1 h. The samples were then allowed to cool naturally.

### Synthesis of GO and LGO

GO and LGO were synthesized using a modified Hummers method. First, 10 g of GP and LGP was mixed with 380 mL of sulfuric acid at 5 °C, followed by the slow addition of 50 g of KMnO_4_ while maintaining the solution temperature below 12 °C. The mixture was stirred for 12 h and cooled to 5 °C. Then, DIW (2500 mL) was slowly added while maintaining the temperature below 10 °C, followed by the addition of 30 mL of hydrogen peroxide. The resulting GO and LGO were washed with 1 m HCl (2000 mL) via vacuum filtration. The obtained GO and LGO powder were dispersed in acetone, washed to remove the unreacted materials and metal impurities, dried, and redispersed in distilled water. Centrifugation was then performed to remove impurities from the GO and LGO solution.

### Preparation of LGP Paste and Ink

To prepare the LGP paste and ink, LGP was mixed with a 15 wt.% CMC aqueous solution at a CMC:LGP mass ratio of 1.5:8.5, homogenized through overhead stirring, and sonicated for 1 h to form a paste for 3D printing. For preparing the ink, the 3D printing paste was diluted two‐fold with water.

### Preparation of TEM Samples

Cross‐sectional LGP samples for TEM observation were prepared using an ultramicrotome. The LGP sampled was embedded and cured in epoxy, sectioned into 100 nm thick slices at a 45° angle using a diamond cutter, and placed on a 200 mesh carbon grid. TEM observations were performed at an accelerating voltage of 200 kV. Fast Fourier transforms (FFTs) were obtained using the GATAN software to quantify the *d*‐spacing of the graphene sheets within the LGP.

### Sample Preparation for 2D Raman Analysis

2D Raman spectroscopy (DXR3xi) was used to investigate the chemical states of LGP and LGO. To minimize noise, LGP flakes and LGO monolayers were coated onto a SiO_2_ substrate. The laser wavelength was 532 nm, the power was maintained at 10 mW, and the exposure time was 2.0 s (0.50 Hz).

### Characterization

The topography of the LGO nanosheets was examined using AFM (XE‐70, Park Systems, Korea) in the tapping mode. The thicknesses of the LGO single sheets were measured using analysis software (XEI, Park Systems). The morphologies of the LGP and LGO nanosheet were characterized through SEM (Hitachi S4800, Japan) at 15 kV and 10 µA. XPS was performed using a theta‐probe‐based system (Al‐*Kα* radiation; Thermo Fisher Scientific Inc., USA). Raman spectroscopy (NRS‐3100, Jasco, UK) with an Nd laser (532 nm) was performed to investigate the changes in crystallinity at different stabilization and thermal annealing temperatures. XRD was performed using a MiniFlex benchtop X‐ray diffractometer (Rigaku, Japan) using Cu‐*Kα* radiation (*λ* = 1.5418 Å). TGA was performed using an STA 449 F3 Jupiter thermal analyzer (NETZSCH) in the temperature range of 30–800 °C at a heating rate of 5 °C min^−1^ under an Ar atmosphere. Fourier‐transform infrared spectroscopy was performed on a Nicolet iS50 spectrometer (Thermo Fisher Scientific) using the attenuated total reflection technique. The electrothermal properties of the LGP electrical heater were evaluated using a simple copper apparatus. The fibers were fixed to a copper electrode using silver paste. A DC power supply unit (Kikusui PAV320–1.3, Japan) with regulation capability was used to evaluate the electrothermal properties of the LGP heater. The voltage and time were controlled based on the experimental conditions using company‐supplied software (Kikusui Wavy ver. 6.0, Japan), which enabled user‐programmed procedures to supply the voltage. During voltage application, the temperature of the LGP heater was monitored using a thermal imaging camera (ET320, Teledyne FLIR, USA). The photothermal performance was evaluated using a solar simulator (500 W ozone‐free xenon lamp with a power of 1 sun).

### Statistical Analysis

The data processing was performed using Origin Pro software (OriginLab Corp.). The electrical conductivity of pure LDPE‐derived graphite, graphite produced from various LDPE waste sources, natural graphite, and Super P is presented as the average and standard deviation of five measurements per sample.

## Conflict of Interest

The authors declare no conflict of interest.

## Supporting information



Supporting Information

## Data Availability

The data that support the findings of this study are available from the corresponding author upon reasonable request.
