# Supplementary material for: Upcycling Waste Low‐Density Polyethylene into Highly Crystalline Graphite
Source: Adv Sci (Weinh). 2025 Apr 11;12(22):2416978. doi: 10.1002/advs.202416978 (PMC12165018; doi:10.1002/advs.202416978)
Supplement: Supplementary file 1 — Supporting Information [file ADVS-12-2416978-s001.docx]

Supporting Information

**Upcycling Waste Low-Density Polyethylene into Highly Crystalline Graphite**

*Woojae Jeong*, *Haneul Nam*, *Hwansoo Shin*, *Sooin Hwang*, *Junho Lee*, *Dongho Lee, Hyeong Jun Kim,* *Jingi Ahn*, and *Tae Hee Han*^*^

W. Jeong, H. Nam, H. Shin, S. Hwang, Joonho Lee, Dongho Lee, Hyeong Jun Kim, and Prof. T. H. Han

Department of Organic and Nano Engineering

Human-Tech Convergence Program

Research Institute of Industrial Science

Hanyang University

Seoul 04763, Republic of Korea

E-mail: than@hanyang.ac.kr

J. Ahn

Hyundai Motor Company

Materials Research & Engineering Center

Sustainable Materials Research Team

Uiwang 16082, Republic of Korea


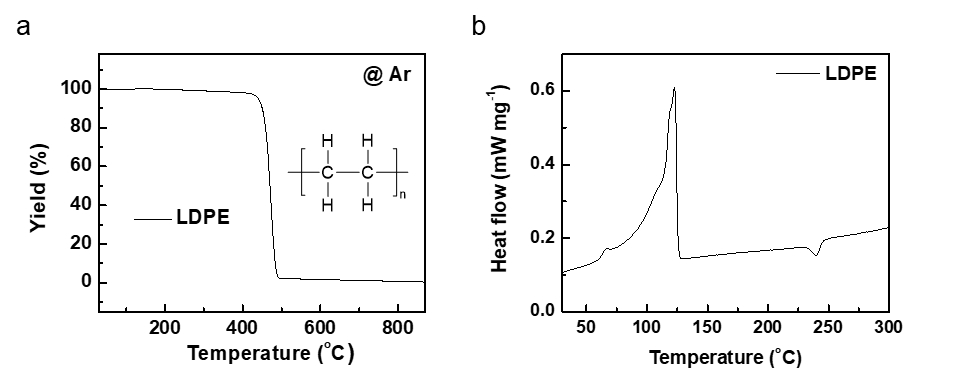


**Figure S1**. (a) TGA spectrum and (b) DSC data of pure LDPE.

|  | **Stabilization process** | ***L_a_***  **(nm)** | ***L_c_***  **(nm)** |
| --- | --- | --- | --- |
| **LDPE** | H_2_SO_4_ hydrothermal | 0.82 | 0.75 |
|  | HN hydrothermal | 1.19 | 0.96 |
|  | Air condition annealing | 0.93 | 0.88 |
| **HDPE** | H_2_SO_4_ hydrothermal | 0.73 | 0.66 |
|  | HN hydrothermal | 0.92 | 0.79 |
|  | Air condition annealing | 0.75 | 0.71 |
| **PP** | H_2_SO_4_ hydrothermal | 0.63 | 0.53 |
|  | HN hydrothermal | 0.64 | 0.52 |
|  | Air condition annealing | 1.03 | 0.83 |

**Table S1**. *L_a_* and *L_c_* values of LDPE, HDPE, and PP after hydrothermal treatment with H₂SO₄ and HN, and air annealing at 250 ℃, followed by thermal annealing at 800 ℃ in Ar condition. (Processing temperature: 250 ℃)


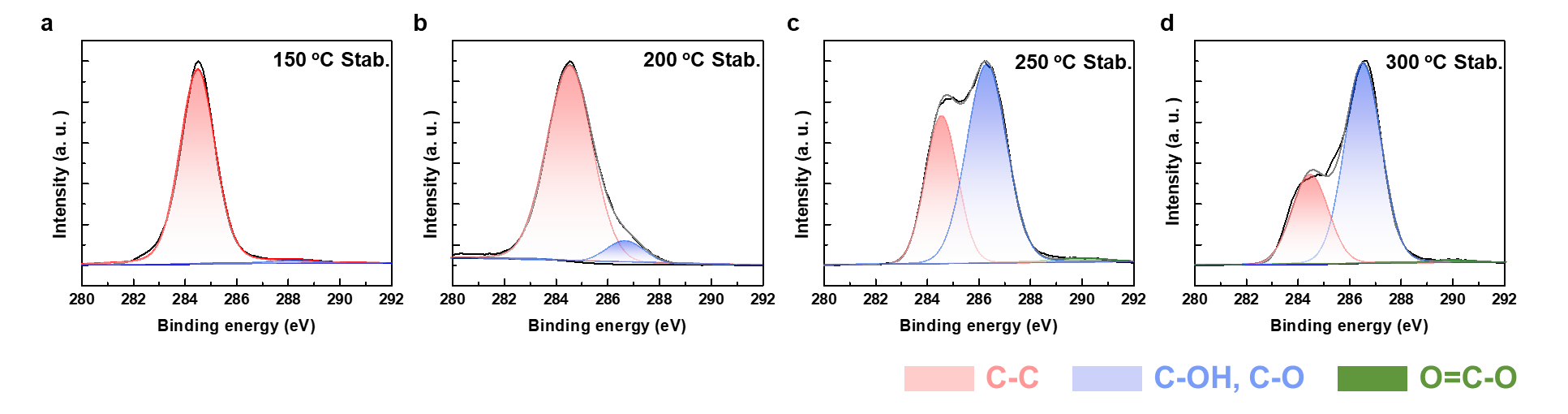


**Figure S2.** XPS C 1s spectra of LDPE samples stabilized at various HN-stabilization temperatures: (a) 150, (b) 200, (c) 250, and (d) 300 ℃.

**Table S2**: Table of elemental analysis results by temperature for HN-stabilization treatment.

| Stabilization temperature (℃) | Ultimate analysis (wt. %) | | | |
| --- | --- | --- | --- | --- |
|  | O | C | H | N |
| **150** | 2.54 | 83.25 | 14.08 | 0.12 |
| **200** | 7.96 | 80.86 | 11.06 | 0.12 |
| **250** | 11.36 | 78.56 | 9.88 | 0.20 |
| **300** | 15.44 | 75.05 | 9.39 | 0.12 |

**Figure S3**. FT-IR spectra of LDPE samples HN-stabilized at 300 and 320 ℃.


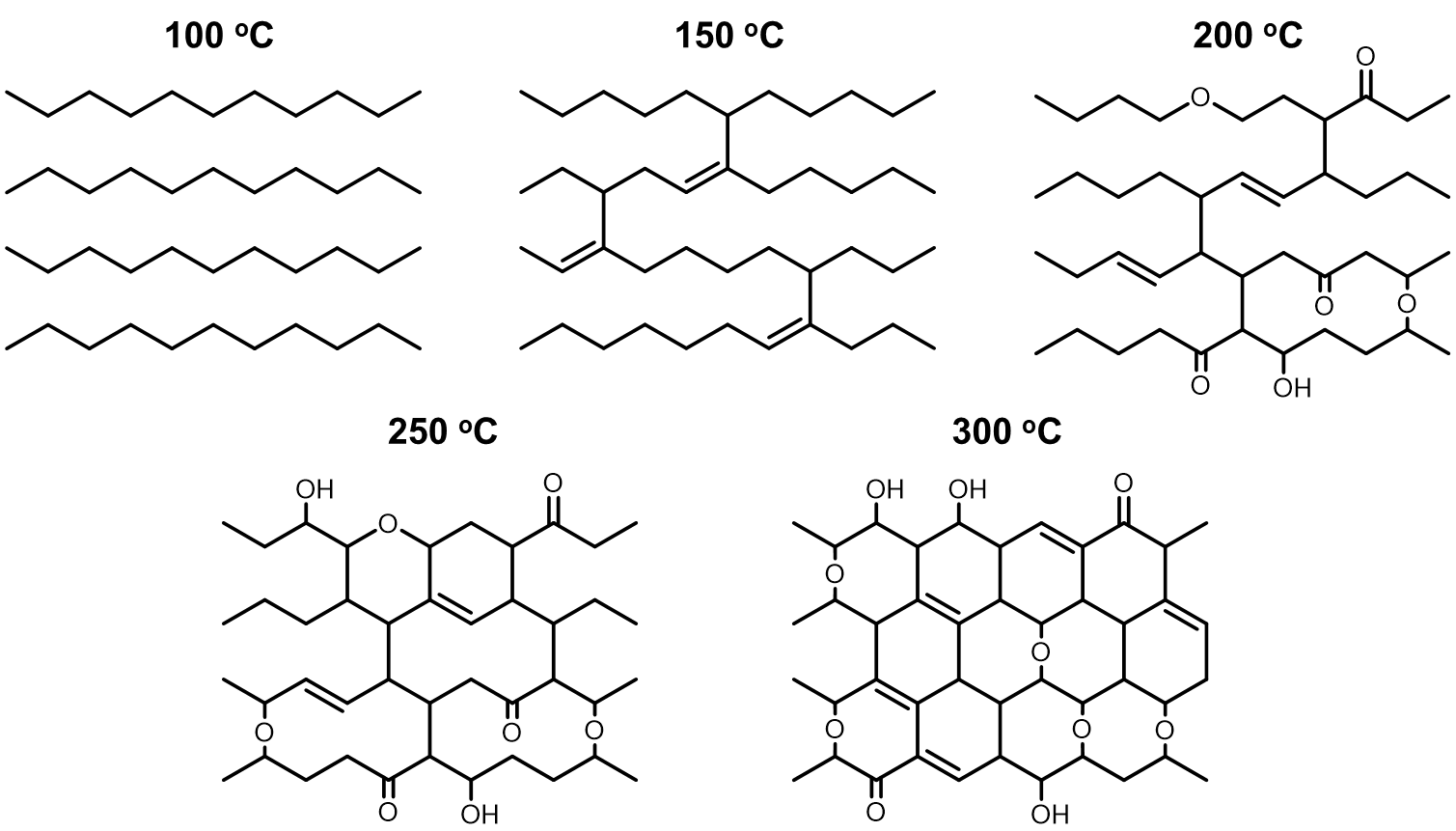


**Figure S4.** Proposed Chemical structural transformation of LDPE chains into cyclized polyaromatic moieties through HN-stabilization.

**Figure S5**. XRD spectrum of HN-LDPE annealed at 1600, 2200, and 2700 ℃.

**Figure S6.** (a) AFM topography and line profile (white dashed line), and (b) SEM images of LGO synthesized from LGP.


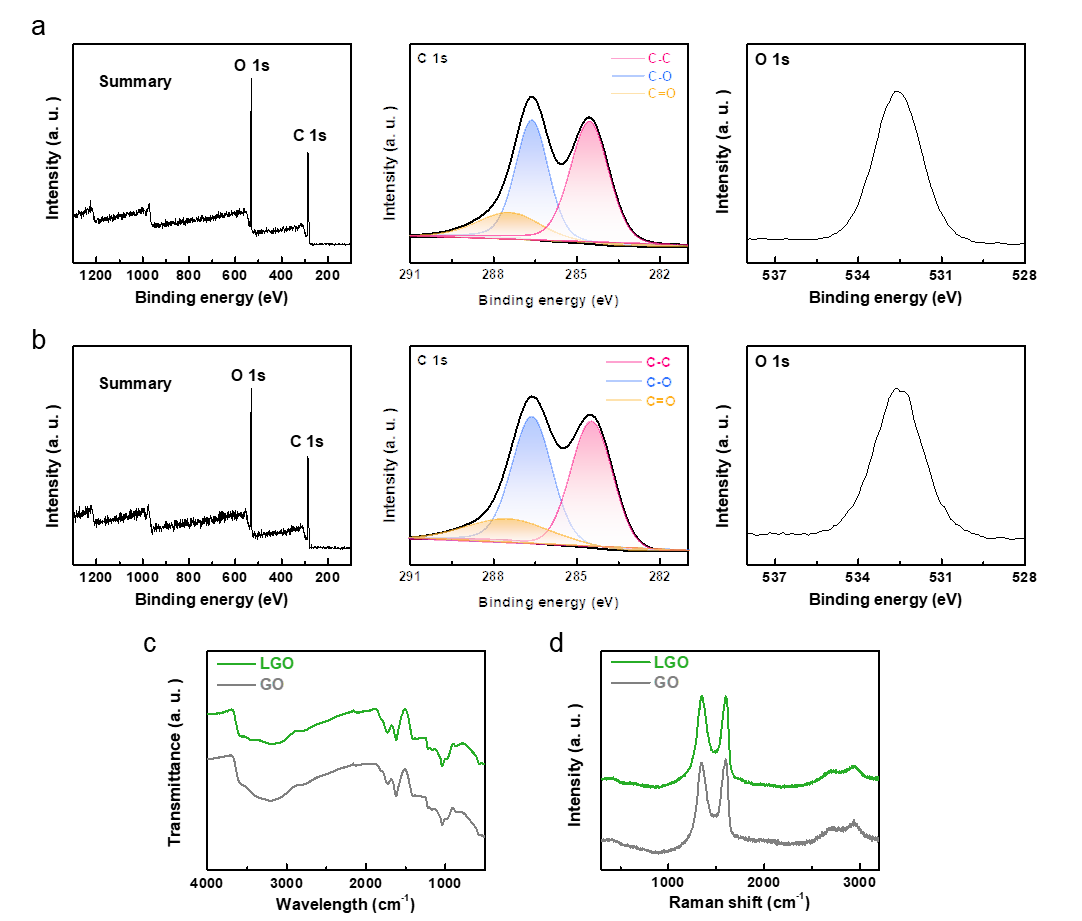


**Figure S7**. XPS spectra of (a) LGO and (b) GO, (c) FT-IR spectra, and (d) Raman spectra.

**Figure S8**. Photograph of waste LDPE products (from left to right: disposable gloves, cling film, and mulch film).

**Figure S9**. Photograph of unreacted materials after LGO production from waste LDPE products.
